# Power in the ‘Organisation’: A Soft Systems Perspective

**DOI:** 10.1007/s11213-020-09541-w

**Published:** 2020-09-19

**Authors:** Frank Stowell

**Affiliations:** Systems and Information Systems Group School of Computing, University of Portsmouth, Portsmouth, PO110DN Hampshire England

**Keywords:** Organisational Change, Soft Systems, Power, Commodity, PEArL

## Abstract

All systems must adapt in order to survive, this is as true for a business organisation as any other system. A business exists in a turbulent environment and in order to maintain its relationship with its environment its managers have to adapt it to the circumstances. The effect of the present pandemic is an example with some staff working from home, active blended learning in the education sector and social distancing have all created an urgency to accommodate the unprecedented consequence of the situation. To effect the necessary changes that these circumstances have generated means exercising some form of power to change operating procedures. Change creates uncertainty, the threat of the reallocation of resources, delegated power, redundancies and a change in group relationships. This produces a feeling of insecurity in those within the organisation often resulting in resistance to the proposals in an attempt to maintain the status quo. They are faced with adapting or resisting to these changes. Whilst systems models of organisational behavior provide ideas about organizing and managing an enterprise these are of limited value because of the unpredictability of change. The ubiquity of communication technologies and the rise of virtual methods of working add to the pressure for change creating a climate of anxiety. Organisational power can no longer be framed by the measures once taken for granted. To this end I adopt a soft systems perspective to explore the impact of change upon an organisation and how those within react as they attempt to cope with its impact.

## The approach taken in this paper

Businesses exist in a turbulent environment and to survive must adjust and adapt to changing circumstances. This situation is heightened presently by the challenge’s resultant upon the drastic measures that have been taken Nationally (and internationally) to lessen the impact of Covid-19. For example, an average of 46% of the working population across the UK have been working from home (ONS April 2020). It seems likely that that many would like to continue with this practice. Such changes in operating procedures mean that businesses have had to rapidly adapt their working practices and those unable to adapt will not recover. At the time of writing it is not yet clear what the long-term effects will be upon the operational process of organizations, but there is likely to be re-structuring and the introduction of new working practices and the loss of employment for many. These are significant changes and for those that remain there will be a feeling of insecurity and loss of confidence. It is axiomatic that instituting organisational change means an exercise of power either to implement it or to resist it. Past examples of such major changes to working practices suggest that this will result in some form of resistance from those most affected. Managing the changes also necessitates new ways of managing the insecurity and rebuilding confidence.

In this paper I will first consider what is meant by ‘organisation’ especially in the light of the present measures and the influence of information technology. I will then consider organisational power from a soft Systems perspective (e.g. Stowell 2020), how it is exercised and the difference between ‘soft’ and ‘hard’ or formal power. This is not to disregard respected commentators who take a different perspective such as those that subscribe to Foucault’s discussion of truth and power (Foucault 1994, pp.111–133) instead, I take Husserl’s notion of phenomenology, as the basis of our understanding and reflect upon Gadamer’s notion of ‘truth and method’ as a practical means of implementing these ideas. I consider Gadamer’s belief in the importance of discussion and, what he refers to as, a fusion of horizons, will provide a useful way of highlighting anxieties felt by those who feel threatened by change. This view is contrary to that of Foucault et al. who argue that discussion is oppressive and discourse a means of governing social groups, instead I take the view espoused by Gadamer, that our understanding is improved by engaging with ‘others’ in a manner that allows each participant to express, unhindered, their opinion about the ‘thing’ in question. I am aware of the differences that Derrida and Gadamer had in their interpretation of Heidegger’s ideas (and de facto Husserl) and these are worthy of exploring further, but it is not appropriate to expand upon these differences within a paper of this length My intention is to explore how Husserl’s thinking combined with Gadamer’s belief in discussion might be valuable in ‘managing’ the effects of organisational power. To this end I will also describe one way that these ideas could be put into action by calling upon the mnemonic PEArL (Champion and Stowell 2001).

## Introduction

In 2020 the world experienced a devastating pandemic that has and is changing interactions both within and between nations. All nations have been affected some devastatingly so. The Globalization of economies and businesses that support them have had to find new ways of adapting if they are to survive. It is self-evident that to meet these challenges changing the way that they operate is necessary. Those that work within will have to adapt to the new processes and new ways of working. Change brings with it insecurity which often manifests in some form of resistance and the employment of ‘power’ by those affected in an attempt to adjust the situation into one where they feel less threatened.

There is no universal and accepted definition of organisational power in the literature. Descriptions of power vary^1^ but a common thread shows them to be model heavy (often systems models), but these are too unwieldy to be helpful. In many instances power is treated as if it exists in the real world and as such, in an equally tangible world, can be investigated through the use of a model or measured by observation. The study of power is not the neat incision into society that a model implies, it is much more involved than that. The enlightening discovery came from the very founder of the concept of politics^2^; Aristotle. He recognized that human beings are unavoidably different and in any social grouping different interests will be being pursued. (e.g. Aristotle 1981*,* p.108, Checkland and Scholes 1990, p.50). If this is not to disrupt or break the collectivity, there needs to be arrangements, generally agreed to and used, which enable power play to be contained: It will always happen. Furthermore, the development and ubiquity of ICT has acted to change the notion of ‘organization’. The modern organization is able to combine strategy makers from different locations across the world via virtual teams in order to facilitate around-the-clock work. These teams are geographically and culturally dispersed and are important mechanisms for business organizations seeking to leverage scarce resources across geographic and other boundaries (Ebrahim et al. 2009). A ‘working from home’ pattern grown out of measures arising from containing the spread of the virus has created a new dynamic in the way an organisation operates. This complexity makes a once-and-for-all model of ‘organization’ problematical and trying to make sense of how it operates challenging.

Researchers no longer think about an organisation in terms of an entity defined by its physical premises, organization charts or models but as, communities of practice or social ‘systems’ (e.g. Wenger and Snyder 2000; Van Wyk 2003, p.43). Despite the changes brought about by technology the prime purpose of those that manage them remains, which is to maintain the relationship of the ‘system’ to its environment.^3^ In doing this it means the organisation is in a constant state of flux as it adapts to change.

The casual use of the noun ‘organisation’ as a description of a particular organized structure is specious, a *falso amico,* as each ‘organisation’ can have so many different meanings. When I refer to organisation here it is non-specific and should be thought of as a ‘System’.^4^ I will begin this paper with a summary of what I deem to be an organisation and then on to the core of this paper ‘organisational’ Power.

## The difficulty of defining ‘Organization’

The difficulty of defining an organisation is not new. An organisation is an abstraction, an intellectual construct and as a consequence difficult to produce a once and for all definition because everyone has a general uninspected idea of what it is. For example, a simple, but vacuous, definition of organisation is, *it is an organisation because we say it is*. Despite the apparent absurdity of this definition it has some validity because, from an observer’s point of view, a collective of any kind will be recognised by something that gives it a meaning for them and could be described as an organisation. I suggest an organisation can be defined by (i) the formal manifestation identified by the name by which it is publicly known and (ii) the informal definition based upon personal (subjective) experience; these are not mutually exclusive. An organisation is a ‘system’ of some kind recognised by an ‘emergent property’ and described by an observer as having a purpose.

Those seeking to understand the working of an ‘organisation’ is confronted by the challenge of making sense of its processes. This is no trivial undertaking as the way in which it functions has many dimensions so thinking in terms of a fixed model or a ‘solution’ based upon an ‘ideal’ case from past success is deficient.^5^ One valuable source of information comes from the programme of action research undertaken at the University of Lancaster^6^ that lasted for over three decades. This valuable research provided important insights into organisational inquiry. It was found that beneath the multiple concepts that can be used to describe organisation there are three elements:

i) A social collectivity, displaying
ii) Multiple world-views and world-views changing over time.
iii) Would-be purposeful activity

Although it is arguable that since that time and in the light of recent events this does not go far enough, never-the less the research surfaced many aspects that remain true today despite the changes that are taking place. For example, the researchers realized that each organisation was unique and as such any investigation had to consider alternative ways to gain understanding. The relationships between those that manage an organisation and those that undertake its operations define its character and resilience, which makes a sharp definition exceptionally difficult to pin down and yet to the observer it is a tangible entity. Individuals are there for different reasons, which are, as Vickers^7^observed, constantly revised or confirmed and as such will have different meanings for each person. One alternative is to think in terms of a ‘system. Taking a cue from the Lancaster project I find it worthwhile thinking in terms of a ‘system’ to [*do something*].…, or a ‘*something*’ System’. It encourages me and others that might be involved to think afresh about the situation of interest. An example of the value of this approach is given by a discussion with some engineers in a manufacturing company. The coordinator was pleasantly surprised to hear them suggest naming it a *materials conversion system* rather than a factory. Their suggestion provided a fruitful basis on which to rethink the function of their manufacturing processes. Thinking in systemic terms helps to gain a greater understanding of ‘*what it is’;* its essence.^8^ Thinking about something as a System in this way gives ‘shape’ to the phenomenon with which we are concerned; it helps the observer to gain an appreciation of the situation in its entirety.

## ‘Organisational’ Insecurity

An organisation is a system set up for a particular purpose and managed through its formal structures from which policy and operational processes emanate. But it is the informal infrastructures through which individuals interpret and operationalize the formal ‘rules’ that are important if these rules are to work. The formal operational structure operates through a form of partnership with the informal networks, and it is the mutual interpretation of management policies (rules) from which develops a kind of understanding that evolves from the degree of tolerance, the give and take, between the formal and informal activities. Awareness of the way actors interact and how individuals make their bonding and transactional decisions is fundamental to the successful management of an enterprise. But change brings with it the threat of the reallocation of resources, delegated power, changes to organizational boundaries and a change in group relationships. It threatens on the one hand a defence of territory and on the other opportunities to make territorial gains. Zaleznik points out *‘...the problems of organizational life involve the dangers associated with the losses of power; the uncertainties are legion especially in the recognition that there is no one best way to organize and distribute power, and yet any individual must make a commitment to some form of organisation’*^9^(ibid, 1970).

Change upsets the mutual security experienced by each agency and their acceptance of unwritten rules is disrupted, disturbing the ‘balance’ between the way these networks operate. As each boundary alters it brings a feeling of insecurity and may lead to a breakdown of the mutual understanding existing between the various individuals and groups within. A breakdown in trust is exemplified when staff work to rule, often creating a major disruption to the operation of the enterprise, sometimes bringing it to a halt.^10^

Understanding of such complex relationships is no trivial undertaking. Not all those that work there will share the same targets but accede to its strictures all the time it suits their main, and often undisclosed purpose. Tönnies, for example, describes two kinds of organisational relationships; there is a community (*Gemeinschaft)* and there is an organisation that is bound together in a contractual sense where the social relationships are individualistic and impersonal. This he called *Gesellschaft.* Tönnies says that “…*Nothing happens in Gesellschaft that is more important for the individual’s wider group than it is for himself. On the contrary, everyone is out for himself alone and living in a state of tension against everyone else*” (Harris 2009, p.52). Checkland and Holwell made a similar point when they suggested that an organisation is not “…*simply a rational machine whose members willingly combine together to pursue organisational goals*” (ibid, 1998, p.80). Such an instrumental relationship to which they refer is in contrast to the informal relationship we feel as part of our family or the close-knit community to which we belong (*Gemeinschaft).* Here we have relationships built on a sense of belonging and being a part of a group of likeminded individuals. Husserl describes such groups as being ‘communalized’. These communities he describes as comprising individuals sharing the same sense of nature. Experience of the real world suggest that harmony within any community rarely exists in perpetuity, an ‘organisation’ is no exception. The turbulent environment experienced by each organisation suggests stability to be a transient condition (Vickers 1983b, C.9). To accept this view, and experience proposes it has some credibility, I suggest it better assume that whilst its members appear to willingly agree with a management strategy this may be only when it serves their particular ends.

## Turbulent Environment

A business ‘system’ exists in a turbulent environment and those that manage it have to deal with conflicting demands, including utilizing existing assets such as infrastructures, resources, expertise and personal loyalties.^11^ These relationships can be troubled when new policies, working procedures or the introduction of technology act to disrupt the concord existing between the infrastructures. This can create anxiety as it may seem to those involved that what is taking place is more than changes to operating procedures, it also signals some form of revision of rules. A classic example of such a situation was the so-called print dispute^12^ in 1986–87. The News International dispute was precipitated by the attempt to introduce ‘new technology’ into the newspaper printing trade and was strongly resisted by the printing trade unions. After failed negotiations and increasingly fraught confrontations the employer found success by enforcing employment legislation through the high courts. The dispute affected not just the way newspapers are printed but, some commentators maintained, the erosion of civil liberties. There were 1260 people arrested and severe restrictions on travel in the Wapping area and a significant cost to the taxpayers. There were many twists and turns in this affair, some confrontational, some underhand and some an outright struggle for power. It is not possible in this brief reference to do justice to them all and a reader interested in a systemic account of the use and abuse of power (on both sides) might find some satisfaction in Stowell’s account.

A more recent example of the effect of changing the policy was the junior doctors^13^ industrial action with the NHS between 2015 and 2016. The government wanted to make changes to the medic’s contracts, which impacted their pay and working hours. The new contracts (new rules) were partly designed to make it cheaper to roster extra doctors on at weekends. Although basic pay would increase the changes to the contracts meant that payment for unsociable hours would be curbed and doctors would find they would be working more often over weekends which, under the existing contract, would have led to extra pay. The dispute led to a series of strikes, including a total withdrawal of labour, by trainees; the first walkouts they had ever staged. The dispute dragged on for some months with periodic stoppages and some medical procedures halted. There was little dialogue between ministers and the union and the dispute generated ill feeling within the profession with each side blaming the other for the failure to reach an agreement. Mark Britnell, head of global health for KPMG after working for the NHS for 20 years, is on record of saying *‘…It makes no sense, Junior doctors will be crucial in making the changes the NHS needs to cope with all the challenges it faces, but they will be demoralised. You can’t improve services without bringing the staff with you.* (Triggle 2016 and BMJ 2016; 352:i1346). The dispute finally ended after four years with an improved pay rise and working conditions. The chair of the BMA’s junior doctors committee said the agreement had come about *‘through a new collaborative, constructive negotiation process that has learned from the mistakes of the past’* (Campbell 2019).

Whilst both of the cases referred to above have been written about and analysed it is doubtful that even if access was granted to the detailed accounts of the discussions that took place behind closed doors a ‘true’ account could be written. An objective analysis from the literature, especially after so long, is unlikely to provide a satisfactory explanation as the disputes were so emotionally charged. I acknowledge that a positivist approach could help shed light of some of the functional issues such as the law, the opportunity costs of installing the technology (in the case of the print dispute) and the cost of an all-out strike. But closer involvement will reveal that there were various strategies employed by each side of the argument. Moreover, there were different systems involved, including union power, political will, financial investment, individual and group motivations and so on. A closer inspection of each of these disputes suggest it was unlikely to have produced an amicable settlement because the changes represented a new set of rules and de facto a new ‘system’. To satisfy the aspirations of the staff and management there needs to be an interchange of information between its membership, to enable some degree of self-determination. This is important as the informal structure is bound up in the value systems existing within the organisation and when there is a change of ‘rules’ staff credibility is more likely to be gained if the staff and those wishing to change the ‘system’ are a part of the discussions. But it would be careless not to acknowledge the dramatic changes that information technology has made to organizational forms and with these changes the nature of interactions between individuals and groups.^14^ Those that make up an organisation may not share the same cultural experiences, business ethos or even language. Organisational power can no longer be framed by the measures once taken for granted.

## The Organisation and the Consequences of Changing the Rules

One area of agreement that all commentators share is that the introduction of a change in working practices or in the structure of the organisation, both commonplace today, may cause the ‘community’ to react, either to comply or resist the proposals. Those affected will accept or adopt strategies to overcome or resist the change. Rather than risk challenging formal power individuals may choose to use their soft power to try to modify these policies if they threaten the status quo.

Even a cursory examination of the News International and the NHS disputes show that there is a myriad of reasons standing behind the confrontations. These range from loss of income to loss of face but at its base was the change of rules. The established ‘systems’ of the past that relied upon the tacit agreement between participants was thrown into disarray. The trust that existed (even where this was grudging) was broken and what emerged was a contest between those that wanted to change the system and those that did not.

The interactions between various groups within any ‘system’ can be likened to a ‘game’, not in the sense of a trivial game of amusement or a set of mathematical probabilities, but as a situation in which the individual (the player) is absorbed by the structure of the situation itself. All the time this structure is stable then the player is contented with letting things stand. But when the rules of the game change so do the relationships within the game. To this end I have adopted Gadamer’s concept of play (Gadamer 2004, p.102–109) as a metaphor for the way that participants, or players, interact within the system.

Whilst Gadamer begins by writing of ‘play’ as a representation of a form of art he develops the idea further describing playing as being a natural process. He says it is pure self-presentation. He writes *‘...The structure of the play absorbs the player into itself, and thus frees him from the burden of taking initiative…’* (ibid, p.105). Initially individuals ‘submit’ themselves to the culture^15^ of the organisation and go along with the prevailing policies because it is the path of least resistance. Individuals joining the organization are often willing to accommodate the ‘rules of the game’ as it saves them taking responsibility, particularly when it satisfies their needs.

The rules of the game can be compared to the operational procedures, the formal rules of the organisation and the way that the rules are interpreted are responsible for the character of the game itself. The ambiance that exists is created from the experience and trust that the participants have for each other. It is axiomatic then that the atmosphere that exists within the organisation is determined by the way the rules are applied. The interpretation of the rules by those involved can be thought of as the unwritten rules built over time as part of an evolving game, a sort of give and take between those that want to implement change and those that fear its consequences. But the consensus shared between players can be upset by change and a subsequent adjustment of the rules. For those within, any change in the rules of the game can shake their very existence, so a sort of cat and mouse game ensues.

Gadamer says that in order for there to be a game there has to be something, someone, others with which the ‘player’ responds with a countermove. The ‘participants’ in the ‘game’ particularly if known to each other, may anticipate the way in which the rules will be applied and consequently determine the atmosphere of the game, or working environment. A trouble-free working environment is the result of the experience and trust built between those involved. But within any organization there are power structures and groups that combine for a variety of reasons and when threatened by change, resist. A change in ‘team’ (community) membership means participants may no longer be able to anticipate how the rules might be applied or what strategies they might introduce. Change brings about the threat of a reallocation of resources and a change in group relationships. Coping with change, ‘…*represents either an adjustment to the situation or an adjustment of the situation’* (Cox 1987, p.7).

An incautious shift in practices may break the harmony that exists between the activities that are designed to achieve a specific purpose and the social systems within the communities that enable them. A social system involves multiple interacting perceptions of reality (Checkland and Poulter 2006). In addition to the recognition of expertise, within each social community there is an informal bonding between individuals that is difficult to recognize by an outside observer. Individuals may (or may not) belong to a loose cultural community fashioned from ‘communalized’ living and doing. As such this ‘community’ is barred to anyone from another community entering in relation to theirs. Our understanding of the ‘world’ is in respect of our surrounding world or culture, our consciousness of the world is created by and from ‘things’ which are around us. It is those ‘things’ which are of relevance to ‘*me*’, which I experience that forms the basis of understanding of the world and my relationship to it. Because our experience is *subjective* and is ever changing such communities can only be fully appreciated by those who belong. Changes to the formal and informal structures of that community creates a feeling of insecurity. Changing the rules may be interpreted as a strategy to enable a particular aspiration such as personal gain, or simply survival to manifest, it changes the dynamic of the community. Until a new relationship has been built up it is reasonable to suppose that participants will adopt a defensive stance until they can establish or preserve their position. Each member will make assumptions based upon their knowledge of similar situations that may create disharmony and lead on to some form of action.

## Disharmony and Organisational Power

Each individual gives the ‘organisation’ power over themselves in return for some kind of reward, material and physical. Being a member means giving up some freedom of action and commitment to management policies (rules). Membership constrains as well as enables (e.g. Vickers 1983b, p.96) because sanctions are part of the armory of those who manage, and it is not always in their interests to allow too great a divergence from their policies. Omisore and Nweke observe that diversity affects ‘…*internal group processes…’* and impedes performance and ‘*…Although it might bring more creativity to problem solving and product development, it could impede implementation.* (ibid, 2014, p.166 & p.177). In other words, if management policies are to be implemented control has to be exercised. This implies some form of power being applied to counter unwanted circumstances.

The literature on power has many different forms and one issue that emerges is its importance upon decision-making. Marx and Engels described theories of power as a ‘trick in three acts’, theorists, ideologists and philosophers’ who provided the ruling classes with a self-serving belief in them.^16^ In the influential text ‘The Anatomy of Power’ (1983) Galbraith describes organisational power as a conduit for the submission of workers. This control is achieved by punishment, reward/compensation or it is conditional relying upon persuasion and correspondence of values. Capra defines an organization as one that is designed specifically to organize the distribution of power, (Capra and Luisi 2014, p.313), implying that within its structure exists an in-built set of covenants to ensure compliance.

In the 1980’s Pfeffer suggested that power is neglected because the concept itself is problematic and troublesome, he went on to say that ‘…organizations are systems in which influence processes play an important part...’ (Pfeffer 1981, pp.1–2). More recently he refers to the importance of being accepted by the community (ibid, 2010, p.2). He refers to the various attributes each of us has, such as experience, loyalty and thirst for power. But I think that Pfeffer is more interested in outcomes and the attributes that he associates with success (observed from a particular point of reference) of these strategies. There are many influential and feted accounts of organisational power developed from this perspective and I acknowledge Pfeffer’s examples of individual strategies for dominance but what I am highlighting is not ambition, but the kind of situation that ordinary individuals might face in their working environment. In such cases the use of personal power is more about job security than malevolent plotting. For example, Raven’s (1992) reworked ‘Power Interaction Model’, *“…describes the agent as a rational decision maker who weighs various costs and benefits of the power bases available to him or her before invoking one of them to influence the target.’*(Mundate and Bennebroek Gravenhorst 2003, p.7). The observation highlights a view that individuals have various kinds of power at their disposal and they select one or more they think appropriate to influence their ‘target’. It is recognition of the dynamic of a social situation in which the actors are in a kind of negotiation with each other. Patočka writes, “…*when surrounded by mere things we are alone, whereas the presence of the other acts on us as a specific stimulus, we feel observed, at the mercy of the other’s understanding*.” (ibid, 2016, p.94). What we understand is based upon our own experience of the world which may be at variance with what the ‘other’ meant to convey; there is room for misinterpretation and the possibility of unintended consequences.

## Organisational Power in an Intangible world

I have suggested power is a neglected concept with no universally agreed definition. An examination of the literature does little to satisfy a researcher looking for a tangible model in an intangible world. It reveals a mixture of views, that includes management science, social science, psychology, anthropology and politics, much is based upon a reductionist belief in objectivity and facts.

I believe that it is also a mistake to assume a binary perspective about the way that people interact. As Zaleznik pointed out that a *‘…realistic coalition matches formal authority and competence with the emotional commitments necessary to establish and maintain the coalition’* (ibid, 1970, p.7)*.* This kind of *‘real world’* relationship requires constant negotiation and compromise between members of the community*.* This emphasizes relationships, which are sometimes harmonious and sometimes fractious, as a coalition between those that manage a ‘system’ and those that implement their policies.

An alternative to the positivist^17^ way of understanding organisation power is interpretivism, where subjectivity is recognised as fundamental to our understanding. Writers such as John Van Maanen^18^ and Bruno Latour speak of power as being the result of experience of its effect. These writers describe power as an intangible, difficult to pin down force, a kind of buffer between the world built upon personal experience and past reactions. Their view is of the phenomenal world deriving its structure from the nature of the mind that perceives it. It is imaginary, transcendental. The idea relies upon the ‘shared’ but often unstated understanding between those involved that such a force exists, enough to make someone comply. It is not a fixed entity but something that can be called upon when needed. This application of power comes from roles that have the ability to reward and sanction as well as power derived from personal attributes such as expertise, charisma and personal attraction. This kind of power cannot be stored.

Latour, something of a polymath, is generally thought of as philosopher, anthropologist and sociologist but thinking of some of Latour’s ideas as transcendental has merit. For example, he speaks of power in an abstract way embracing inanimate objects. He refers to Durkheim’s view of the importance of ‘resources’ that bind a clan together. Examples include such resources as flags, colours, tattoos and so on. Such objects are, he says, integral to membership; they are symbols of ‘belonging’ and through the shared meaning of certain symbols provide a source of power for that group and that moment in time. This is the basis of Actor Network Theory (ANT). He argues that ‘*power is not something you can hoard or possess, it is something that has to be made’*. The use of power is relational and not necessarily shared by all because, ‘… *The obedience to an order given by someone would require the alignment of all people concerned by it who would assent faithfully without adding or subtracting anything. Such a situation is highly improbable. The chances are that the order has been modified and composed by many different people who slowly turned it into something completely different as they sought to achieve their own goals.’* (Latour 1986). From this perspective power is contextual and temporal and becomes less useful as power increases or decreases (ibid, 1986, p265). He speaks of power in terms of a ‘primary and secondary’ mechanism. Primary mechanism as a composition made by other people and secondary mechanism *attributed* by another.

Latour’s argument is persuasive, but I find myself questioning if it fully explains the ebb and flow of influences that emerge in any social ‘hub’. A key feature of ANT is that it is based upon ‘objects’ as ‘vessels of power’, yet there are instances where power is exercised with no apparent tangible item. Such instances as beauty, emotion all have the potential to transform one state into something other than it at first appeared. Beauty will influence an observer in different ways. For example, for some an arachnid represents the evolution of one of nature’s creatures to be admired, for others it is regarded as a threat. Whilst the former might belong to a ‘community’ of like-minded naturists the latter extends across a wide and disparate proportion of the population, often defying logical explanation. We learn of the effect of power to influence outcomes from experience. As Gadamer reminds me, ‘…*power cannot be known or measured in terms of its expressions, but only experienced as an indwelling*’ (ibid, 2004, p.202).

Power is more than its expression, power possesses potentiality. Individuals will be aware of the different ways that power is exercised. It is not just what is said, but the way it is said often accompanied by physical mannerisms that has the potential to convey a meaning to others. Here we have, for me, two important aspects of power. The first is that the recognition of power is gained from experience and the second gained from the way in which it is expressed. Fully understanding either or both of these is subjective and I feel it is a mistake for an observer to attribute ‘power’ to an object because what we see and hear is understood by each observer in terms of their experience of something they consider to be similar; it is noetic.^19^ I believe that there is a kind of power that cannot easily be defined but it is readily experienced,^20^ it is a form of power, a soft power, a force that is something existing ‘within’. It is immanent, restricted entirely to the mind or a given domain; it is internal and subjective. In this sense it seems clear to me that this, *soft* power, is best understood by each of those who experience it in action and who are aware of its consequences.

## Soft Power and when we use it

Any form of power that is perceived as being used outside the accepted value systems of the community will be seen as illegitimate and invoke a reaction. The act of introducing change will involve the use of power, either the formal power vested in authority or as a form of resistance^21^ . As Capra points out *‘…people do not resist change; they resist having change imposed*…’ (ibid 2003, p.87). Experience shows that individuals and groups exercise a form of power which is outside the parameters of the formal structure or their particular role or position. This I call soft power.

Soft power, relies upon a shared, often unstated, understanding between those involved that such a ‘force’ exists, and it is only by experience do we gain an idea of this kind of power. Soft power is associated with an individual rather than the role they may or may not occupy. This power is derived from a personal attribute that is conferred upon someone by a third person and is *subjective* (similar to Latours secondary mechanism). Its use can be significant and as researchers have observed, ‘*…the use of soft power in social groups is often used to great effect in discussions and it at times ‘trumps’ that of formal power’* (e.g. See Cooray 2010; Hart 2014). The acknowledgement that such power exists is also to concede it to be subjective and contextual. It is not possible to define what it is in such a way that it can be applied to another situation. It relies upon the acknowledgement and agreement of the community in which it is exercised.

Putting to one side examples that rely upon the indoctrination of individuals into a particular world view, such as blind support for a football team or to an ideology (this is a topic requiring a paper of its own), what remains seems to be another sort of power. This is a ‘personal’ power and applied in situations where an individual might feel threatened by change and forms some sort of defence or in some cases it is used as a form of self-promotion. But power need not be used as a force for suppression and coercion it can be used in a supportive way for an individual or group. As Jary and Jary observed, ‘*Although power …is often seen in negative terms, as involving coercion and conflicts of interest...‘power’ can also be seen in more positive terms, as enabling.’* (ibid, 1995, p.513). Power can be creative and inspirational, we can recognise the power of love, the power of poetry and the power of laughter as positive uses of power. Experience teaches us to recognize this kind of power but it is difficult to define, highlighting our appreciation of the world is both subjective and temporal and any attempt, by an observer, to categorise each perception of power will be illusory.

To imagine that *soft* power can be defined would be to accept that the ‘attribute’ contained within the definition has been identified and objectively ‘tested’ and an unprejudiced description that satisfies the principle of pure evidence. This flies in the face of the notion of soft power. *Soft* power is based upon things that mean something to the ‘community’ and accepted by them *at that time* and in that context. Each ‘community’ will share or be willing to tradeoff the worth of certain things that provides them with a sense of security. To understand how ‘soft’ power is used and what value it has within any group situation requires a subjective appreciation of what is taking place and not one constrained by the need to ‘prove the model^22^‘.

We all possess ‘soft’ power and soon learn surreptitious ways to ‘get our own way’. It is something we recognize and learn to use from an early age (e.g. Piaget’s acclaimed studies on cognitive development). We learn to apply unobtrusive strategies in order to find ways to help us ‘cope’ with *our* situation including calling upon personal attributes such as reputation, personality, esteem to help us to be accepted within our community (e.g. see Zaleznik 1970; Stowell 1989a, 2018; Checkland and Poulter 2006).

Whilst there is some form of continuity as we transfer from one familiar community to another eventually, we join an alien ‘domain’. Joining a new community opens up a new set of circumstances and we enter a community with little knowledge of others or the norms within that community. In order to feel secure individuals’, seek to exercise some form of control over their environment. We develop a way of coping and either fit in or try to change things to make us feel more secure, a sort of transformation of intent takes place as individuals and groups attempt to gain a greater amount of control. I suggest thinking of soft power in terms of a metaphor^23^ reflects its abstruse nature and might prove useful.

## A Metaphor for soft Power

A simple definition of the use of power adopted in this paper is ‘The ability to ‘convert’ someone to adopt a course of action at variance with their original inclination’. But defining in absolute terms what soft power *is* is difficult, if not impossible. Whilst ‘hard’ power can be seen in action e.g. orders or sanctions soft power can only be gauged by its effect. Soft power is intangible, and it is its effect that gives the *impression* of power, which leaves an observer unable to ‘put their finger’ on what it was that changed the direction. It is something possessed by us all, but not necessarily used. Everyone has the *potential* to exercise their soft power. The difficulty in identifying what it is, the inquirer is often left with ‘footprints’ of power rather than something that can be explained to others, you either understand when it is used, or you miss it. I believe thinking in terms of a metaphor might provide a helpful means of understanding its effects. It avoids the necessity of defining each instance where it has been observed and keeps faith with the subjectivity of its interpretation, much as calling something a system is valuable in that it gives shape to the phenomenon with which we are concerned. I suggest thinking in terms of the metaphor *Commodity* provides a ‘trigger’ to explore, through discussion, an interpretation or explanation of what it was that influenced a change in direction. I have proposed using this metaphor as a way of offering a neutral account of the perceived inputs (soft power) of individuals and groups in which attempts are made to translate a situation into something else.

Referring to soft power as a *commodity* facilitates free discussion in the spirit of Gadamer’s ‘Fusion of Horizons’^24^ where participants work to exchange knowledge rather than seek to impose a particular opinion, (Gadamer 2004, p.304; Stowell and Welch 2012, pp.147–148). Using the metaphor Commodity is a way of highlighting the effect of soft power. In this sense ‘Commodities’ are culturally based and will vary between individuals and social situations, social groups and between organisations. Importantly, its ‘value’ is more readily understood within context. It is within context of group interaction through language and other cues that can only be fully understood, or even recognised, that the power embedded in social exchange emerges. Patočka, discussing the phenomenon of language, remarks, *‘…individual subjects seek to assert themselves, and since the action of each individual always involves broader or narrower communities, linguistic communication is a primary instrument of the perpetually renewed social combat*.” (Patočka 2016, pp.91–92). But it is not confined to verbal communication, often non-verbal cues can also be used to influence the result.

Thinking in general terms about the ‘process’ of power and hence the notion of using a metaphor has proved useful. Champion and Stowell say that by *‘Applying the ‘power as a commodity’ the metaphor facilitates asking how power has been expressed within the situation and how people intend to use and maintain these ‘commodities’* (ibid, 2001, p.9). By thinking in terms of a commodity the researcher could gain two advantages. First, a better, more holistic, understanding of the situation and second, the metaphor can be used to discuss the effects of power within the group. Managed sympathetically ‘Commodity’ opens the way for a wider range of discussions to take place that may bring out hidden but imagined manifestations of power. As Gadamer reminds us, *‘A man who is disguised does not want to be recognised, but instead to appear as someone else…we merely pretend, act a part, and create an impression.’* (ibid, 2004, p.111).

Recognizing the use of a ‘commodity’ (or commodities) provides an opportunity of giving weight to the opinion within the context of the way in which this ‘commodity’ has been expressed. By surfacing ‘commodities of power’ enables a stream of analysis to be created. We can ask, what are these commodities? How are they obtained, exercised, defended, suffused or limited, relinquished? It is this idea that underpins the Analysis One, Two and Three stages incorporated within SSM. In Analysis Three Checkland questions the nature of power, and asks *‘What is Power? It works with the fact that everyone who participates in the life of social grouping quickly acquires a sense of what you have to do to influence people*.’ (Checkland 1999, p.A20).

## Metaphor in use

Stowell (1989b, p.151) suggested that the metaphor can be considered at three levels; conceptual, propositional and discursive level. In practice the participants attempt to understand the reason and motive behind the way the ‘commodities’ are being applied. At the propositional level they can attempt to understand the purpose and intent with which individuals are using their soft power and at the discursive level observations about the formalization of statements concerning action.

Providing a list of examples of ‘commodities’ is counterproductive as the way in which soft power is used is specific to a particular group and situation. Whilst we acknowledge, as Ryle remarks, ‘*Understanding is still psychological divining*…’ (Ryle 1990, p.52), as individuals we call upon our experiences and understanding of the culture of which we are a part, to make sense of interactions between participants. The lack of examples is less an indication of the poverty of the idea, but more a reflection of the kind of thinking that needs to ‘hypothesize’ and make a clear connection between the example and the situation. There is a contradiction between this idea and the mindset that demands such a logical connectivity, in that this kind of thinking implies some form of proof. Commodities of power are both tangible and intangible. The notion of controlling power seems to relate to many different attributes which are as diverse as charisma, physical strength, education, beauty, status, property ownership and as examples of a negative effect, withdrawal of affection, lack of communication, exclusion, physical threat.

It is important to recognize that ‘Commodities’ change through time because they are personal, cultural and temporal. For example, in a work setting an individual may use the value placed upon their skills or their position within the organisation. Within a domestic setting the same individual may capitalize upon ‘emotional commodities. In a social setting they may rely upon personality or physical appearance. The possession of a number of different commodities, which we all have, gives individuals the possibility of exercising some control over an unsettling situation. Clearly different ‘commodities’ may be employed to fit different situations. In situations where individual livelihood is at stake, then those that identify with the problem may act in unison and combine to resist a common threat e.g. withdrawal of labour or ‘work to rule’. In other situations, they may act independently.

The way that different commodities of power are employed might alter the outcome of a discussion. Observations of situations where change was taking place shows examples of the way that the employment of the soft power can act to give advantage to those with the skills to use them.

- Illustration 1: Stowell (1989a) describes a situation that manifested itself during a project to change the working practices in the commercial department of a manufacturing company. A new export contracts manager had been appointed and became involved in the project that had been on-going for several months. The project had been developed into one of participation and of shared ownership by all involved (the commercial department had 50 members, ibid., pp.161). At his first meeting the manager, who had apparently enjoyed a reputation in his previous company for a particular style of management, joined some of the group meetings. The export contracts controller seemed to want to exploit his commodities of power, described by Stowell, as [i] his expertise and [ii] his forthright opinions, *‘He attempted to rearrange the situation in order to make himself a valued asset but he failed to recognise the culture of his new enterprise; he appeared to want the organisation to change rather than accept there to be a new situation. He seemed to be unaware of the culture of his new company’* (ibid, p.268). His attempts to exercise control over the project could be thought of in terms of commodities of power. But his ‘power’ was based on a reputation that was not recognised nor valued by the staff in the commercial department of his new company. His attempts to denigrate the project had exactly the reverse effect upon the staff that he intended. By criticizing the project, he had unwittingly challenged the integrity of the staff who, by this time, were driving the project. The staff saw the new manager as attempting to undermine their efforts; he was considered an alien.

The project provided many instances of the use of *commodities* in a positive sense as well as being used through veiled threats for those who wished to maintain the status quo. For example, the managing director preferred to use his charms and personality to control the situation rather than use his formal power. He had initiated the project as a means of appeasing his managers by bringing in someone from outside the company. Although he wished to change the ways of working he chose to do this without seemingly giving any one manager the power over the others and at the same time appearing to have no direct control over outcomes himself. But he could delay or speed up things by sheer force of personality whilst not appearing to be exercising his formal authority. This approach was in contrast to that adopted by the Sales Director who attempted to maintain the status quo by making sure everyone knew of his contacts within headquarters staff (formal authority) and by nurturing his personal relationships with certain staff who opposed the changes (soft power) as a means of displaying the scope of the potential resistance to ideas that he did not share (ibid, pp.266–267).

- Illustration 2: Checkland and Poulter (2006) in *Learning for Action* (2006, pp.35–37) provide an example of using the notion of commodity as part of Soft Systems Methodology Analysis 3 (Political). They describe a situation where two managers in a consultancy company were being interviewed together. The conversation between them became confrontational at which point one manager said, “…you are NKT, I’m KT”. Company insiders would have recognised this shorthand as Knew Tom and Never Knew Tom. The significance being those who knew the recently deceased charismatic founder of the company, Tom, took on his reflected glory whereas the opinions of those that did not know him did not carry the same credibility. Clearly this distinction, highlighted by the notion of commodity, reveals an informal, but important, hierarchy. The example also serves to illustrate the way that commodities will change over time. In this case as the memory and influence of the founder fades so too will the “power” of those who chose to use “KT” as a commodity will find its influence will also have faded

- Illustration 3: In two workshops facilitated by Bednar and Stowell, A. in 2011 they found the mnemonic a useful way of helping participants to appreciate the way in which individuals react when they meet for the first time. For example, Stowell. A., (Social Services workshop 2008) was able to recognise the actions of some of the group and the way that they used their assumed status from their formal role in their organisation to try to exert influence (commodity). She noted that this strategy had little effect upon those not connected with the same organisation and was soon recognised and abandoned by the individual concerned. In one workshop she noted that ‘*The group appeared at ease and willing to discuss conflicting views. They accommodated each other by either changing terminology to accommodate a group understanding or agreed to have two different subsystems’* (July 9th^,^ 2008). In the second of the reported workshops there was no evidence of attempts to exercise control over the other participants during group work, as Bednar reported (July 8th^,^ 2008) *‘Informal powers were low key and while some may have been used I believe that this was more personality trait than intention. I would argue that members went out of their way to try to avoid imposing their beliefs on others during this session*.’ (Interestingly Bednar’s reflection about personality trait is a good example of a commodity of power as, in this instance, someone used their personality to good effect at times). On this point it is worth noting that participants might use other ways of trying to influence outcomes

- Illustration 4: In 2014 Hart set up a series of workshops to help her research ‘knowledge transfer’. The workshops were organised in an institution whose prime function was to provide advice to Government on a variety of issues surrounding defence. In this institution staff usually moved on to a different role every 2–3 years consequently retaining knowledge within the organisation was operationally important. This meant that unless the ‘knowledge’ of the individuals was passed on to their replacement they had to effectively start again. As far as Hart’s research was concerned this was an ideal situation in which to gain understanding about the importance and method of transferring knowledge. The workshops included various members (and grades) of staff who agreed to share their opinions to help gain understanding of what they thought about knowledge transfer. Staff were willing to take part with many giving up their free time to join the discussions.

The participants were aware of the relationship between knowledge and its association to personal reputation. They acknowledged that there was a desire by some individuals to maintain their reputation as experts that could act to frustrate knowledge sharing. Hart observed that the possession of tacit knowledge was used by some staff as a commodity of power contributing to an individual’s reputation and offered the possibility of financial reward, e.g. a consultancy role. It seemed clear that this attitude would also affect wider sharing behaviour, but no one would admit to using this ‘commodity’ but acknowledged it existed. (Hart 2014, p.14). There seemed a consensus in the group that the hoarding of knowledge was a further expression of implicit knowledge and every possibility that subject experts had. They ‘guarded’ their knowledge, consciously or unconsciously, this strategy to ‘protect’ their knowledge, she commented, could be seen as an example of a commodity of power (Hart 2014, p14).

Harts study coincided with a time of organizational change. At least one participant, a senior manager of the knowledge services department, did not attend all meetings because he was located in a different part of the country, but importantly, was due to transfer to this establishment within a few months. When present at the meetings he seemed threatened by the discussions (Hart 2014, p.165). It transpired that he had developed a knowledge resources ‘system’ and it is reasonable to assume that he expected to transfer the way he ran his present unit into the new location (Hart 2014, p.87, p.132, p.155). Hart observed that in each session, despite the meetings being set up to explore all aspects of knowledge transfer with no predefined outcome, he seemed to be at pains to ensure that the other participants were aware of his ‘vast’ experience. He saw his task to encourage staff in the operating department to provide his department with ‘knowledge’, a view that was at variance with the opinion of the operating department manager. It should be borne in mind that this research exercise was investigating the influences affecting knowledge sharing, yet this individual attempted to steer the discussion to his ends (Hart 2014, p.155).

Hart was aware of the notion of commodity and was conscious of the way that individuals sought to apply their soft power. Thinking in this way gave context to the discussions and added insight and richness to her understanding of the situation of concern and its outcome.

- Illustration 5: Cooray, in her research into a city library reflected the use of soft power writes that *‘The experiences during the group sessions at the Portsmouth City Central Library show a close affinity with the informal power relations discussed by* Stowell *(*2000*)* *and Checkland (*Checkland and Poulter 2006*) than the formal power relations discussed by critical theorists, since in several instances individuals used commodities in order to exert power and control*’ (Cooray 2010, pp.130). In a later study exploring how ‘commodity’ could be applied in virtual team discussions, Cooray observed that ‘…in this exercise the more technically proficient participants seemed to use their expertise as a way of influencing others. The less technically able were hesitant creating the impression that they were happy to leave this aspect to others. (N.B. It could also be an example of the use of soft power; the implication being a form of ready-made excuse if things went wrong’ Stowell and Cooray 2017)

## A useful Mnemonic: PEArL

Thinking of power as an abstract form described by ‘commodity’ has proved useful in inquiries, but I acknowledge it is not always easy to introduce it into a ‘real world’ situation. For example, Cooray found that it was quite challenging for the clients to distinguish between formal power and informal power relations. To address such concerns, I proposed using the mnemonic PEArL. Cooray found this suggestion to be useful and writes *“…with the incorporation of the notion of power within the mnemonic PEArL we found that it goes some way to address the difficulty and has proved to be a useful suggestion.*..’ (Cooray 2010, p.144).

The notion of PEArL emerges in a paper by Champion and Stowell (2001) in which they propose that the mnemonic helps the inquirer to appreciate the character of the inquiry. PEArL enables the researcher and clients to be explicit about who is included and who is excluded from the situation of interest. By thinking of ‘**A**uthority’ and ‘**r**elationships’ as ‘hard’ or ‘soft’ power provides insight into the connections between participants. Being sensitive to ‘A’ and ‘r’ allows the practitioner/participants to ask how is power expressed in this situation; they can ask what are the ‘Commodities’ that signal influences between the participants. By gaining and appreciation of the way in which soft, or the commodity of power, emerges helps the researcher, and often the client, to explain the power relationships within the boundary of the situation of interest that are thought to exist (Stowell 1989b; Stowell and Welch 2012 pp.74–76; Checkland 1999). Accounts of PEArL^25^ can be found elsewhere (see footnote) but I provide a brief summary of ‘A’ and ‘r’ below.

### Authority

Any process of inquiry within a social organisational setting will be sanctioned by a person, or group concerned with the situation under investigation. Recording who authorised the inquiry and for what purpose and who administers its implementation is essential when considering if the inquiry has been pertinent to the situation of concern. ‘A’ makes clear who has formal control.

### relationships

The models produced during the inquiry process will reflect the intended relationships to be maintained within any proposed intervention. An examination of these relationships may be useful in questioning any undeclared assumptions and worldviews (Weltanschauungen) held by the participants. These relationships may provide insight into how individual power and control has been dealt with by participants during the inquiry and with their intended intervention. Reflecting upon the intended relationships, what they are, what is expressed, the context and how ‘soft power’ is deployed may help to identify potential areas of conflict.

I have found from the practice that embedding ‘commodity’ (r) within the mnemonic PEArL it becomes a visual and practical way in which to explore the implications of ‘soft’ power. It can help with the understanding of the dynamics of the group where individuals seek to ‘persuade’ others to accept their point of view. The lower case ‘r’ is an attempt to clearly differentiate ‘soft’ power from formal ‘hard power as represented by the upper case ‘A’. By surfacing the use of Soft power we can generate an exchange of ideas as a means of gaining an *Appreciation* of the situation of interest.

## Conclusion

Having grown up in a world which subscribes to the scientific paradigm many find it difficult to look outside a world described according to a causal model into one where reality is established from subjective experience of relationships within the human environment. Even within the Systems community some are more comfortable ‘imposing’ a framework as a means of helping them make sense of what they perceive. Whilst we are finite beings and part of a world surrounded by concrete phenomena, we are also in a world of lived-in experience accessible to all to a varying extent. What we make of our world is shaped by a series of concrete phenomena and impressions formed from our (subjective) experience of it and through the communities to which we belong. The naive world in which we start our life, full of ‘feelings’, instinct, wonder, observations and things we do not understand, is gradually eroded by the seduction of the notion of objectivity. Subjectivity, we are soon taught, is close to mysticism and belongs to the past. Explanation, we learn, is gained through the application of a set of structures, which provide a well-trodden outcome. If this fails then we set about trying to find new paradigms or new ways to explain something which, frankly, cannot be wrapped up into a package to be used until the next time; it does not answer our question.

We are afraid of accepting that our naive insights may be of value. To accept that a simple statement uttered in discussion might have a greater depth for the person uttering may be difficult for those who view the world solely in concrete terms. But the manner in which individuals interact may ring alarm bells amongst the participants. To dismiss such phenomena is to deny things that we have all experienced but have found difficult to express. Thinking in terms of a ‘Commodity’ as a metaphor for power has synergy with Heidegger’s notion of hermeneutic phenomenology, which ‘…*proceeds by means of interpretation*’ (Cerbone 2008, p.29). Commodities are part of a transformation process affecting the position held by each participant in a given situation. Starting positions are transformed by the trading of commodities and by the strategies chosen by participants. By making ‘soft’ power explicit opens up the possibility of a fruitful outcome. But the language and the meanings embedded within the ‘conversations’ can only be fully understood by those who are a part. By allowing participants ‘in’ on the way in which people seek to influence outcomes acts as a means of reminding us that we should stop and think ‘what does this mean’; ‘why are they reacting in this way?’ The point of the idea is to develop participants’ awareness of the potential impact that some interactions might have on shaping the outcome. We can gain insight into the way participants attempt to shape the outcome through their use of language and the nonverbal cues they may use to reinforce their meaning. But the outsider is unlikely to be privy to such richness of communications.

What we are seeking is a method of bringing into public debate a description of those ‘Commodities’ that are used to influence that situation. It is not a means of trying to control those with ‘power’ but a means of disarming the way in which it is used. Power is an integral part of political behaviour and its currency is relative to the degree of dependency upon a given ‘commodity’. Thinking about power in this way provides a means of articulating it in terms that is appropriate to a given organizational culture, which can be understood by those most affected and for all to reflect upon and used as part of the process of gaining an holistic view of the situation.

## Footnotes

1 Researchers such as Dahl 1957; Handy 1976; French and Raven, 1959; Blau and Blau 1964; Debnam 1984; Ryan 1984; Cavanaugh 1984; Katabadze and Parker^1984^; Buchanan and Badham 2008; Latour 1986

2 Politics as a practical science - concerned with the noble action or happiness of the citizens (Fred Miller -fmiller@sppfbg.org

3 See Vickers (1983a) Chapter One for further discussion

4 An open systems (organisation) is a whole in so far as it acts as a whole on its surround Vickers 1983a, p.17.

5 e.g. Checkland, (Checkland 1999, pp.141–146)

6 See Checkland’s Systems Thinking Systems Practice 30 year retrospective for a detailed account of the project.

7 Vickers 1983b, p.67 & c.4)

8 See Husserl 2012, pp.10–11; Majolino 2015, pp.42–45 for further insight.

9 This has some resonance with Tönnies (2009) notion of *Gemeinschaft* and *Gesellschaft*.

10 E.g. 1988: Nurses protest for better pay, http://news.bbc.co.uk/onthisday/hi/dates/stories/february/3/newsid_2525000/2525639.stm; Junior doctors. 2017–18, https://www.bbc.co.uk/news/health-34775980; Southern Rail dispute 2019–2020, https://www.bbc.co.uk/news/uk-england-48068392]

11 See Marabelli and Galliers 2016; Watkins, 2007 for a useful discussion.

12 See Stowell 1989 for a full account of the dispute p.306–351.

13 There was also a dispute with the BMA over changes to consultants’ contracts

14 Vickers (1983b p.48) refers to the speed of development of technology emphasising difficulty of control. Gadamer also points to the political difficulty of countering the ‘opinion’ expressed in the media https://www.youtube.com/watch?v=lm-hZY5W4Ss

15 see Husserl’s discussion on intersubjective communities, Cartesian meditations, pp.131–136, Husserl 1991

16 See Krippendorff 1995, for a discussion of power from a critical theorists perspective.

17 Researchers adopting a positivist assume results can be understood without personal value judgments. Research methods that involve the use of quantitative data and requires the use of the scientific method.

18 For Maanen’s (2015) interesting discussion on Organisational Change (from an ANT perspective) see https://www.youtube.com/watch?v=kAbaIb-NpW0

19 Every intentional act has noetic content; it is the mental process that becomes directed towards the intentionally held object - See Husserl 2012, pp.186–190.

20 Essence- perception of something in its basic form (Eidos); see Husserl 2002, C1; Phenomenological reduction is to attempt to see the ‘essence’ of the ‘thing’ of interest

21 E.g. see Stowell and West 1994, C7.

22 By this I mean a scientific or political definition

23 A thing considered as representative of some other (usually abstract) thing; a symbol; (Shorter Oxford English Dictionary 2007).

24 I take this to mean participants explore the boundary of their understanding and experience.

25 PEArL see Stowell and Welch 2012; Champion and Stowell (2001) and an example of its use in Stowell and Cooray (2017, pp. 258–259)
